# Human Adipose-Tissue Derived Stromal Cells in Combination with Hypoxia Effectively Support *Ex Vivo* Expansion of Cord Blood Haematopoietic Progenitors

**DOI:** 10.1371/journal.pone.0124939

**Published:** 2015-04-28

**Authors:** Elena R. Andreeva, Irina V. Andrianova, Elena V. Sotnezova, Sergey V. Buravkov, Polina I. Bobyleva, Yury A. Romanov, Ludmila B. Buravkova

**Affiliations:** 1 Institute of Biomedical Problems, Russian Academy of Sciences, Moscow, Russia; 2 Institute of Experimental Cardiology, Cardiology Research Center, Moscow, Russia; 3 Faculty of Basic Medicine, Moscow State University, Moscow, Russia; Karolinska Institutet, SWEDEN

## Abstract

The optimisation of haematopoietic stem and progenitor cell expansion is on demand in modern cell therapy. In this work, haematopoietic stem/progenitor cells (HSPCs) have been selected from unmanipulated cord blood mononuclear cells (cbMNCs) due to adhesion to human adipose-tissue derived stromal cells (ASCs) under standard (20%) and tissue-related (5%) oxygen. ASCs efficiently maintained viability and supported further HSPC expansion at 20% and 5% O_2_. During co-culture with ASCs, a new floating population of differently committed HSPCs (HSPCs-1) grew. This suspension was enriched with СD34^+ ^cells up to 6 (20% O_2_) and 8 (5% O_2_) times. Functional analysis of HSPCs-1 revealed cobble-stone area forming cells (CAFCs) and lineage-restricted colony-forming cells (CFCs). The number of CFCs was 1.6 times higher at tissue-related O_2_, than in standard cultivation (20% O_2_). This increase was related to a rise in the number of multipotent precursors - BFU-E, CFU-GEMM and CFU-GM. These changes were at least partly ensured by the increased concentration of MCP-1 and IL-8 at 5% O_2_. In summary, our data demonstrated that human ASCs enables the selection of functionally active HSPCs from unfractionated cbMNCs, the further expansion of which without exogenous cytokines provides enrichment with CD34^+ ^cells. ASCs efficiently support the viability and proliferation of cord blood haematopoietic progenitors of different commitment at standard and tissue-related O_2_ levels at the expense of direct and paracrine cell-to-cell interactions.

## Introduction

Cord blood haematopoietic stem and progenitor cells (cbHSPCs) have attracted considerable interest as a full value alternative to bone marrow HSPCs. The number of cbHSPC transplants is increasing from year to year, but their low number in one cord blood sample is a significant limitation to expanded application of these cells [1]. In this connection, the development of *ex vivo* approaches for cbHSPC amplification and differentiation into certain haematopoietic lineages is a primary goal of cell technologies. The creation of conditions for *ex vivo* expansion of cbHSPCs based on peculiarities of the haematopoietic tissue niche may noticeably improve the expected results.

The importance and necessity of stromal cells for *ex vivo* expansion of haematopoietic cells was reviewed by Dexter et al. in 1984 [2], and others [3,4,5]. As a result of direct intercellular and different soluble mediator-mediated contacts between MSCs and HSCs, the functions of the latter are effectively regulated, such as self-renewal and differentiation.

Until recently, most of such experiments were conducted on stromal and haematopoietic cells isolated from bone marrow [1]. The results of these studies demonstrated that MSCs, applied as a feeder layer, may considerably influence the co-cultured HSCs, in particular, changing the ratio of poorly differentiated and committed progenitors [3,4,6,7]. The advantage of stromal sub-layers to increase the number of primitive progenitors that ensure long-term haematopoiesis reconstitution was demonstrated [3,8]. These data are very important as HSCs expansion in semisolid media results in the predominant proliferation of committed progenitors at the expense of primitive ones [3].

At present, mobilisation of alternative sources of both haematopoietic and stromal cells is being explored increasingly more intensely. For example, expansion of *ex vivo* HSCs from cord blood (cbHSCs) and peripheral blood after stimulation with appropriate cytokines is being actively studied for further application in clinical practice [3,4,9,10]. It was shown that the expansion of cbHSCs, like their bone marrow counterparts, can be efficiently achieved in liquid and semiliquid environments, as well as on different stromal feeders [3,4,10]. Primary MSCs are one of the most attractive types of cells to be used as a feeder *in vitro* [5,11]. The effects of stromal cells on the HSCs supported by them may vary depending on the tissue used for MSC isolation [5,12]. In particular, it was shown that MSCs from the stromal-vascular fraction of adipose tissue (ASCs), unlike other stromal cells, predominantly support proliferation and differentiation of committed haematopoietic progenitors [12,13]. On the contrary, recent studies have demonstrated the advantage of using ASCs for primitive HSC expansion compared to bone marrow MSCs [14,15]. In any case, as ASCs may be easily isolated from the patient’s tissue in sufficient quantity, they are the most attractive among the alternative sources of stromal cells.

One of the most characteristic signs of local haematopoietic milieu is low partial oxygen pressure, which may affect cellular elements and their interaction in the haematopoietic niche. In bone marrow, O_2_ varies from 0% to 4–6%, depending on the distance from the blood vessels [16,17]. O_2_ concentration is known to play an important role in regulation of the state of both haematopoietic and stromal cells [17,18]. With growing understanding of the O_2_ role, increasingly more attention is being paid to elucidation of the peculiarities of MSC function under O_2_ concentration characterised as “physiological hypoxia”, which is similar to tissue-related O_2_ values [19,20]. In our lab, as well as in several other laboratories, it was demonstrated that the permanent expansion of MSCs, including ASCs, at O_2_ close to “physiological hypoxia” considerably influences the realisation of their most important features, contributing to support the non-committed state [21,22]. It can be supposed that low O_2_ may also influence the realisation of MSC stromal function. However, there are still no sufficient experimental data on how reduced O_2_ in the microenvironment influences the *ex vivo* expansion of HSPCs on the stromal sub-layer. We were the first to combine bone marrow MSCs and low O_2_ tension (5%) as *ex vivo* milieu for cbHSPCs. This resulted in the more pronounced formation of haematopoietic colonies and increased production of IL-6, IL-8 as compared to co-culture in standard conditions (20% O_2_) [23]. In the case of hypoxia, improved expansion of CD34^+^HSPCs in the presence of osteoblasts [24] and bone marrow [1,25,26] MSCs was demonstrated.

Thus, the data on *ex vivo* HSPC expansion in the simulated haematopoietic microenvironment at tissue-related low O_2_ and on bone marrow MSCs as a stromal layer provide a reason to conclude that these conditions may considerably modulate HSPC properties.

At present, most studies have implemented purified immuno-selected CD34^+^HSPCs for *ex vivo* expansion. This may lead to artificial depletion of the heterogeneous HSPC population, as this antigen is expressed on only some subsets of HSPCs [27]. To enrich selection of the HSPC population from cord blood, we applied a functional approach at the expense of HSPC adhesion to the ASC layer and further expansion of the adhered HSPCs and their progeny [28]. In this study, we evaluated for the first time the potency of ASCs to support viability and self-renewal/commitment of expanded HSPCs (progeny of cells which were adhered to unmanipulated cbMNCs) at standard (20%) and tissue-related (5%—hypoxia) O_2_.

## Materials and Methods

### Preparation of Cells

Adipose tissue samples were obtained in the frame of Scientific Agreement from multidisciplinary clinic “Souz” (Moscow, Russia) after elective liposuction procedures under local anaesthesia from healthy patients with written informed consent. Adipose stromal cells (ASCs) were isolated from adipose tissue using guidelines specifically approved by Biomedicine Ethics Committee of Institute of Biomedical Problems, Russian Academy of Sciences (Physiology Section of the Russian Bioethics Committee, Russian Federation National Commission for UNESCO, Permit #314/MCK/09/03/13), as previously described [29] with some modifications [30]. Briefly, tissue samples were treated with 0.075% collagenase IA (Sigma-Aldrich, USA). After washing, cells were resuspended in αMEM (Gibco, USA), supplemented with 10% FBS (Hyclone, USA), 250 μg/ml amphotericin, 5μg/ml streptomycin, 5U/ml penicillin, and 2mM glutamine (MP Biomedicals, USA).

Mononuclear cells from umbilical cord blood (cbMNCs) were isolated after written informed consent in the Cord blood bank “Cryocenter” (Moscow, Russia) using guidelines of the License of Federal Service on Surveillance in Healthcare and Social Development (Roszdravnadzor) (Permit #FS#2010/342). Cryopreserved samples of cbMNCs were provided as a part of Scientific Agreement between Cryocenter and Institute of Biomedical Problems.

The study was approved by Biomedicine Ethics Committee of Institute of Biomedical Problems of Russian Academy of Sciences.

### ASCs cell culture

ASCs were divided into two parts after isolation. The first was further expanded in a standard laboratory CO_2_-incubator (Sanyo, Japan) with 5% CO_2_ and 95% air (20% O_2_) (normoxia); the other part was propagated in a multigas incubator (Sanyo) at 5% O_2_, 5% CO_2_, 90% N_2_ (hypoxia). After reaching 70–80% confluence, cells were sub-cultured and 2–4 passages of ASCs were used in experiments.

### ASC/cbMNCs, ASC/cbHSPCs and ASC/cbHSPCs-1 co-culture

To enrich the population of cbHSPCs we used the experimental approach described by us previously [28]. Briefly, confluent (70–80%) ASC layers were pre-formed in 35 mm Petri dishes at 20% and 5% O_2_. Then, cbMNCs (1x10^6^/ml) were seeded on these feeders in RPMI 1640 (Gibco, USA), supplemented with 10% inactivated FBS (Hyclone, USA), 250 μg/ml amphotericin, 5 μg/ml streptomycin, 5 U/ml penicillin, and 2mM glutamine (MP Biomedicals, USA) and co-cultured at the same O_2_ concentrations as ASCs. After 72 hours (Day 3), all floating cbMNCs were carefully washed away. One Petri dish in each set was fixed with cold methanol and the attached cbMNCs were analysed after Giemsa staining of the co-cultures. The other dishes with attached cbHSPCs were further expanded with replenishment of the RPMI 1640 culture medium twice per week. After 96 hours (Day 7) of ASC/cbHSPCs co-culturing, the generation of new non-adherent cell suspension (cbHSPCs-1) was observed. Non-adherent cbHSPCs-1 were harvested, enumerated, and assayed by flow cytometry, seeded in colony-forming cell assays and reseeded on new ASC feeder.

To evaluate the potential of newly-formed cbHSPCs-1, 40x10^3^ cells/ml were transferred onto new pre-formed ASC layers, again at 20% and 5% O_2_ accordingly. After 72 hours (Day 11), the morphological observation of attached and floating cbHSPCs-1 was performed, non-adherent cells were removed and co-cultures were further expanded until Day 14.

### Flow Cytometry

Mouse anti-human monoclonal antibodies for CD45, CD34, CD73, CD90, CD105, (BD Biosciences, USA), CD133/1 (AC133) (Miltenyi-Biotec, Germany) and isotype IgGs, conjugated with FITC or PE were used for ASCs and cbHSPCs immunophenotyping. Necrotic and apoptotic cells were revealed with Annexin V—FITC/PI staining (Immunotech, France). Cells were analysed with Epics XL flow cytometer using the manufacturer’s System II software for data acquisition (listmodes) and their cytometric analysis (histograms) (Beckman Coulter, USA).

### Colony-forming cell (CFC) assay

Floating cbHSPCs-1 were collected after 72 h of co-culture with ASCs and 50x10^3^ cells/ml were plated in methylcellulose-based medium MethoCult H4534 (Stemcell Technologies, USA) according to the manufacturer’s protocol at 20% and 5% O_2_. Colonies were scored after 14 days.

### Cobblestone Area-Forming Cells (CAFCs)

CAFCs were defined as clusters of small, tightly packed cells that were non-refractory when viewed under a phase contrast microscope.

### MultiPlex Flow Cytomix Assay

Chemokine concentration in conditioned medium (CM) after ASC/cbHSPCs co-culture was measured using Human Chemokine 6plex (G-CSF, IL-8, MCP-1, MIG, MIP-1α, MIP-1β) assay (Bender MedSystems, Austria) on FAX Calibur flow cytometer (BD, USA). CELLquest software (BD) was used for data acquisition. The concentration of each cytokine was linearly dependent on fluorescence intensity and was calculated using Standard curves that were generated for each cytokine using Flow Cytomix Pro software (eBioscience, USA).

### Light and scanning electron microscopy (SEM)

Bright-field, phase contrast and NAMC analysis of cultured cells was performed using Nikon Eclipse Ti-U microscope equipped with a Colour Digital Camera DS-Ri1. Images were saved and later processed with NIS-Elements Auto Research software (Nikon Instruments, Japan). For SEM ASCs and ASC/cbHSPCs were prepared routinely: cells were fixed in 2.5% glutaraldehyde in 0.1 M phosphate buffer for 1 h at room temperature, post-fixed in OsO_4_ (1% aqueous solution on 0.1 M phosphate buffer, 1 h at room temperature), and dehydrated in ascending acetone concentrations. Dehydrated specimens were dried at a critical point dryer in liquid CO_2_ in Polaron device (Great Britain) and sputtered with gold (100 nm thick) using ion sputtering technique in Eiko device (Japan). Examination of samples was performed using a Hitachi S-500 scanning electron microscope (Japan), equipped with capture self-made device at accelerating voltage of 25 kV with following resolution: 512X512 pixels, 8 bit/pixel.

### Statistical analysis

All data were derived from at least three independent experiments. The results are presented as mean ± standard error of the mean (M±S.E.M). Comparisons between experimental results were determined by Mann—Whitney test for independent samples; p<0.05 was considered statistically significant.

## Results

### Characterisation of cbHSPCs, attached to ASCs

**Fig 1:**
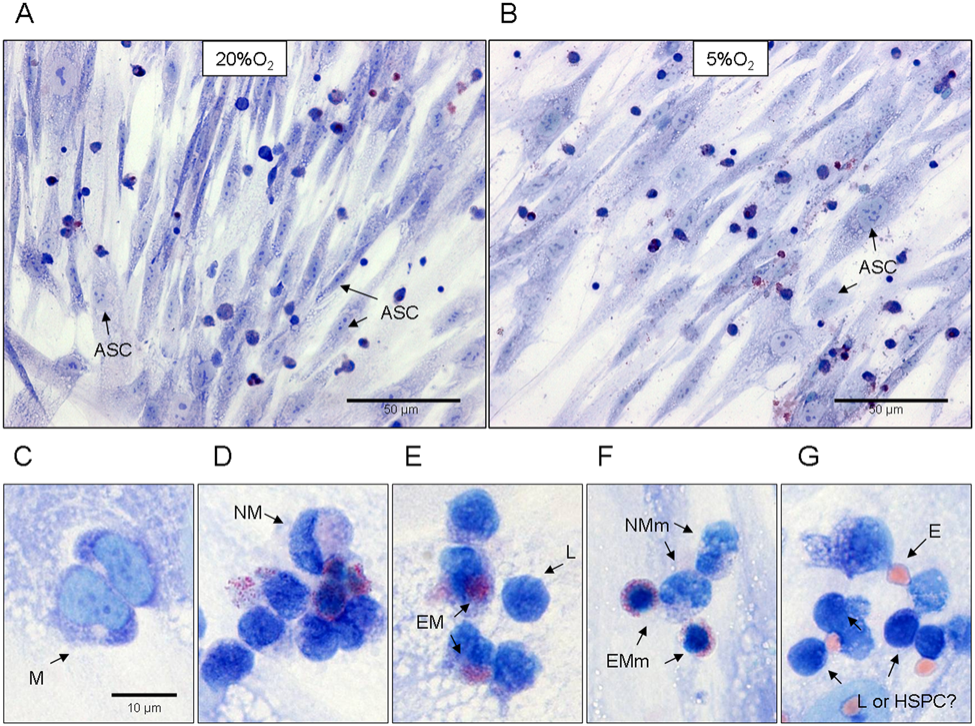
Identification of cbMNCs, attached to ASC layer on Day 3 of co-culturing at 20% O_2_ and 5% O_2_. Unmanipulated cbMNCs were cocultured with ASCs at 20% and 5% O_**2**_. After 72 hours all floating cbMNCs were washed out, ASCs with attached cord blood cells were fixed with cold methanol and stained with May-Grunwald-Giemsa for differentiatial analysis of haematopoietic precursors. A—20% O_2_; B -5% O_2_, general view. Arrows indicate ASCs. Bar—50 um. There were no marked difference between the appearance of co-cultures at different O_**2**_ concentrations. C-G—larger magnification of cbMNCs on ASCs. Bar—10 um. Mature blood cells: M-*monocyte or promonocyte*; E—erythrocyte; L- lymphocyte. cbHSPCs: NM and EM—*neutrophilic myelocyte & eosinophilic myelocyte;* NMm and EMm—*eosinophilic metamyelocyte & neutrophilic metamyelocyte;* HSPCs—morphologically unidentified cells, usually are considered as lymphoid elements.

On Day 3 mature blood-borne cells and myeloid progenitors of different commitments were detected among attached cells with differential Giemsa staining: promonocyte/monocytes, erythrocytes; lymphocytes, neutrophilic and eosinophilic myelocytes, neutrophilic and eosinophilic metamyelocytes (Fig 1A–1G). Besides, cbHSPCs with a basophilic cytoplasm and a large nucleus with 1–2 nucleoli without specific lineage features were demonstrated, which made it possible to refer to them as non-differentiated haematopoietic progenitors (blasts) (Fig 1G).

The adhered cbHSPCs preserved high viability, as shown by flow cytometry after Annexin V—FITC/PI staining. (Table 1).

**Table 1:** Viability of adhered cbHSPCs after 72 hours in co-culture with ASCs.

	*20% O*_*2*_	*5% O*_*2*_
necrotic, %	1±0	1±0
apoptotic, %	13±5	21±2
live,%	86±6	78±15

To evaluate the viability of attached cbHSPCs, ASC/cbHSPC co-cultures were trypsinised, and the cell suspension was stained with the Annexin V—FITC/PI kit according to the manufacturer’s instructions, before being analysed on an Epics XL flow cytometer. For analysis, only cells gated as CD45^+^/CD90^-^ were used. The data are presented as M+S.E.M. (n = 4).

**Fig 2:**
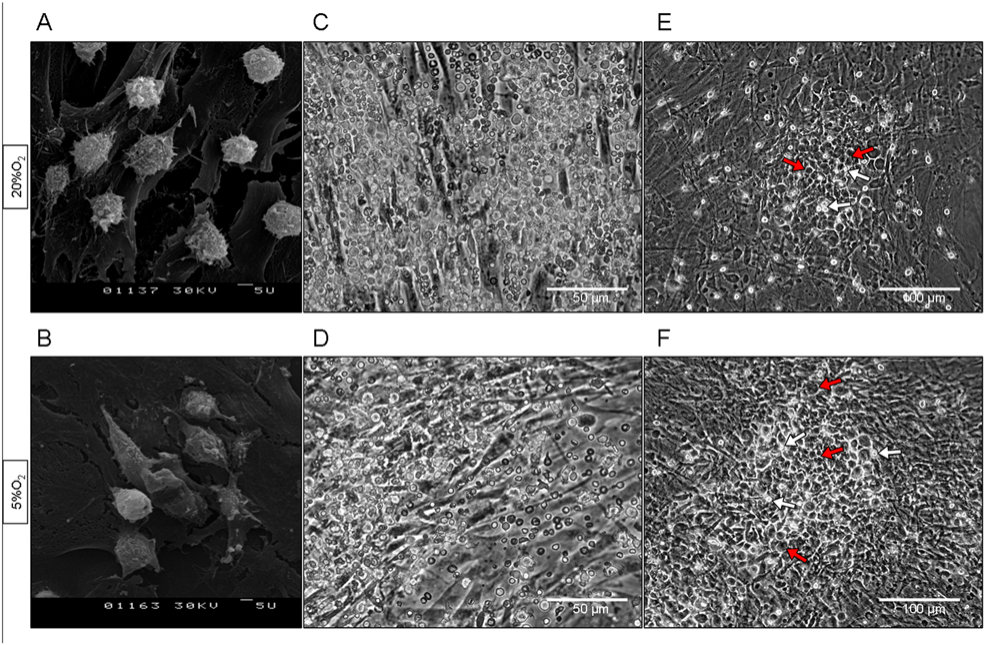
HSPCs at 20% O_2_ and 5% O_2_. cbMNCs were co-cultured with ASCs, washed out after 72 hours and ASCs with adhered cbMNCs-derived HSPCs continued culturing up to 14 days. A, B—single and clustered cbHSPCs, Day 7 in culture. CEM. Bar—5 um. C, D—newly-generated floating cbHSPCs (cbHSPCs-1), Day 7. Phase contrast. Bar—50 um. E, F—CAFC areas in ASC/cbHSPCs co-culture, Day 11. Phase contrast. Bar—100 um. Red arrows indicate phase-dim CAFCs beneath the ASC layer. White arrows indicate the phase-bright cbHSPCs on the ASC surface.

After a further 72–96 hours (Day 7), the single and clustered cbHSPCs were revealed on the ASC surface (Fig 2A and 2B). Besides the adhered cbHSPCs, floating cells were also detected in the co-culture (Fig 2C and 2D). As described previously, these cells are the progeny of the adhered HSPCs proliferating on the stromal layer [31]. These floating cbHSPCs (cbHSPCs-1) were collected and their functional activity was further evaluated. Some cbHSPCs migrated into the space under ASCs, forming cobble-stone like structures, whose area enlarged throughout further culturing (Fig 2E and 2F).

### Characterisation of the newly-formed cbHSPCs-1

#### Number and viability

The number of cbHSPCs-1 was similar at 20% and 5% O_2_ and made up 140±84 and 115±50x10^3^/ml, respectively. The trypan blue exclusion test demonstrated about 90% viable cells both at 20% and 5% O_2_.

#### Immunophenotype

**Fig 3:**
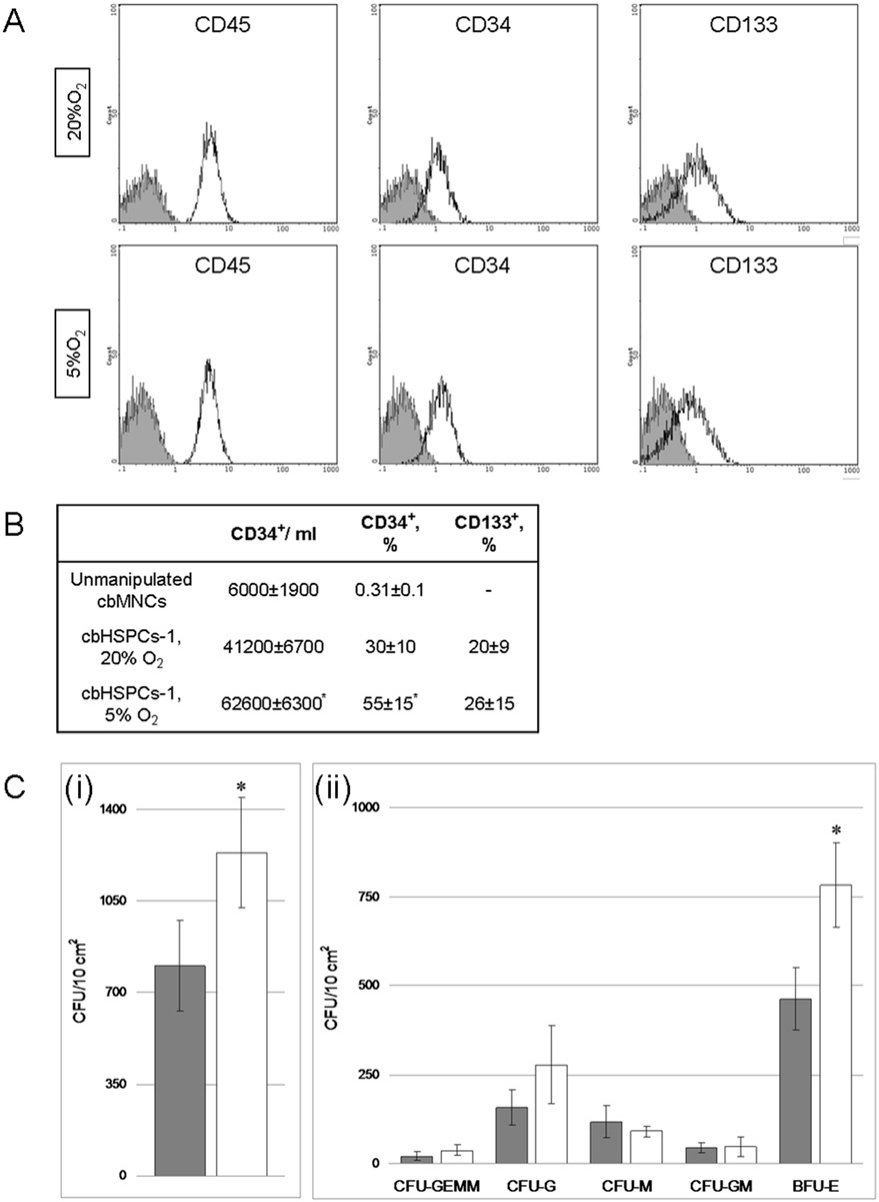
Characteristics of cbHSPCs-1 population at 20% O_2_ and 5% O_2_. Floating cbHSPCs-1 were harvested on Day 7. CD45, CD34 and CD133 positive cells were evaluated with flow cytometry and the number of CFCs was estimated in MetoCult H4534. A. Representative histograms of cbHSPCs immunostaining. Isotypic control—black line, grey fill, positively stained cells—black line. B. Enrichment of cbHSPC-1 population with low differentiated hematopoietic precursors. The data are presented as M±S.E.M (n = 4). *—p<0,05, significant difference from 20% O_**2**_. C. In vitro progenitor assays. (i) Total CFCs number. (ii) The number of early erythroid progenitors (BFU-E), granulocyte-macrophage progenitors (CFU-GM, CFU-G and CFU-M) and multi-potential granulocyte, erythroid, macrophage, megakaryocyte (CFU-GEMM) CFCs. The data are presented as M+S.E.M (n = 5). *—p<0,05, significant difference from 20% O_**2**_.

Over 95% of cbHSPCs-1 cells were bearing CD45 pan-leukocyte antigens. The share of CD34^+^cells among HSPCs-1 significantly increased in comparison with unmanipulated cbMNCs and was higher at 5% O_2_ (p<0.05). Moreover, we failed to identify CD133^+^cells in cbMNCs before expansion, but about 20% of HSPCs-1 were CD133 positive both at 20% and 5% O_2_. The enrichment of HSPCs-1 suspension with undifferentiated CD34+ progenitors as a result of expansion on ASCs made up about 6 and 8 times at 20% and 5% O_2_, respectively (Fig 3A and 3B).

#### Evaluation of CFCs among cbHSPCs-1

To reveal CFCs, HSPCs-1 were grown in methylcellulose-based media for colony-forming unit (CFU) assays (MethoCult H4434) for 14 days and total CFC and lineage-restricted CFC numbers were calculated. The total CFU number of HSPCs-1 at 20% O_2_, was almost 1.6 times lower in comparison with at 5% O_2_ (Fig 3C(i)). More than half of the CFUs were represented by BFU-E, whose numbers were twice as high at 5% O_2_ than at 20% O_2_. Besides, there were more CFU-G and fewer CFU-M at 5% O_2_ (Fig 3C(ii)).

#### cbHSPC-1 reseeding

**Fig 4:**
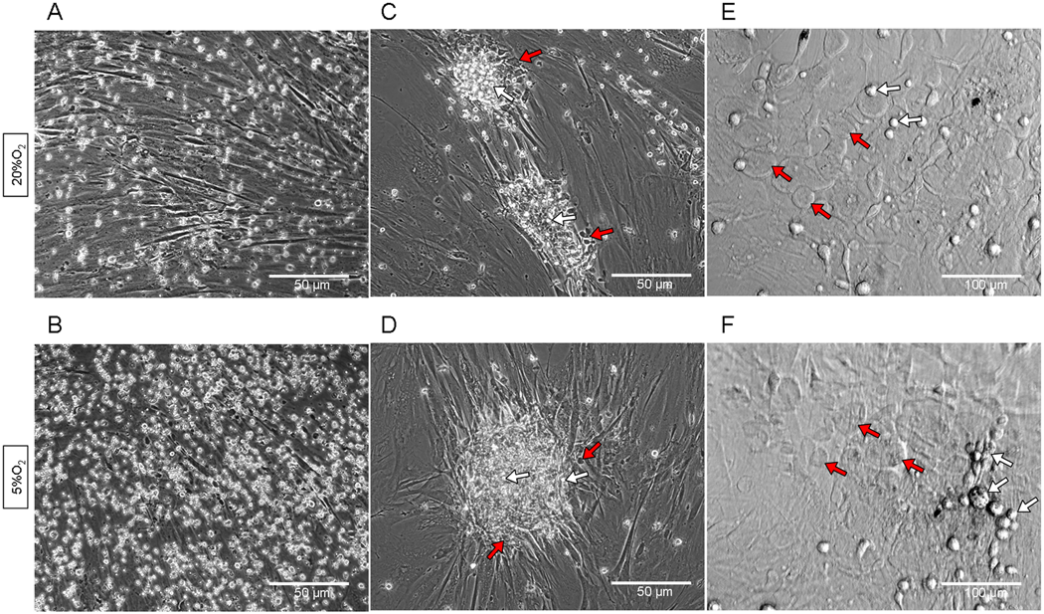
Reseeding of cbHSPCs-1 at 20% O_2_ and 5% O_2_. Floating cbHSPCs-1 were harvested on Day 7 and reseeded on new ASC layer. On Day 11 cbHSPCs-1 were located in three compartments, as was described above for cbMNC-derived cbHSPCs. A, B. An accrued population of floating reseeded cbHSPCs-1 was visible in co-cultures. Phase contrast. Bar—50 um. C, D. Single and clastered phase-bright reseeded cbHSPCs-1 on the ASC surface and phase-dim cbHSPCs-1 beneath ASC layer. Phase contrast. Bar—50 um. White arrows—the phase-bright, red arrows—phase-dim cbHSPCs-1. E, F—CAFC areas in ASC/reseeded cbHSPCs-1 co-culture, Day 14. NAMC contrast. Bar—100 um. Red arrows indicate CAFCs beneath the ASC layer. White arrows—phase-bright cbHSPCs-1 on the ASC surface.

cbHSPCs-1 suspension was plated on the mitomycin C-treated ASC layer at 20% and at 5% O_2_. On Day 11, a floating population of cells was again formed above the attached cbHSPCs-1 (Fig 4A and 4B), single adhered cbHSPCs-1 and newly-formed clusters could be seen, similar to those described above, on the surface of ASCs (Fig 4C and 4D). Besides, after 2 weeks of co-culture, we observed large CAFC-occupied areas under ASCs at both 20% and 5% O_2_, similar to that described above for unmanipulated cbHSPCs (Fig 4E and 4F).

### Paracrine regulation of ASC/cbHSPCs interaction

**Fig 5:**
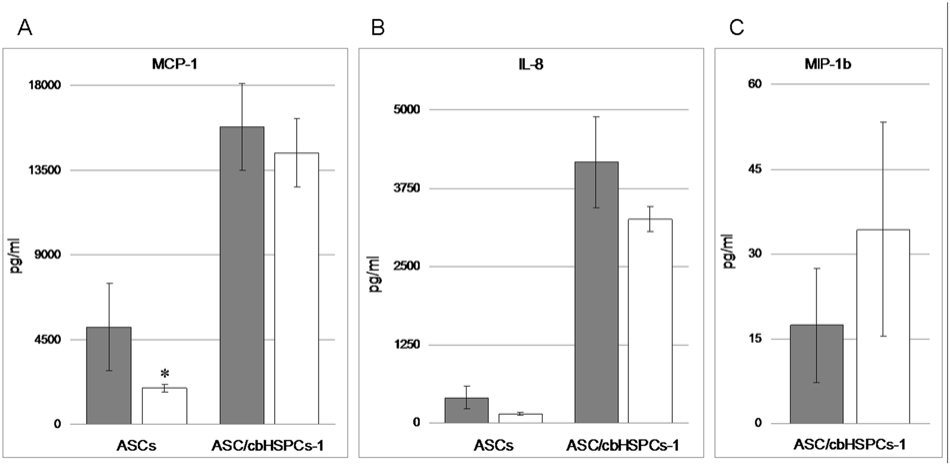
Chemokine production in ASC monoculture and ASC/cbHSPCs-1 co-culture at 20% O_2_ and 5% O_2_. Conditioned medium was collected on Day 7 and chemokine concentration was estimated with Human Chemokine 6plex. In ASC monoculture only MCP-1 (A, left bars) and IL-8 (B, left bars) were detected. In ASC/cbHSPCs-1 co-culture a significant increase of both chemokines was detected (A,B, right bars) Besides, MIP-1β appeared in in co-culture (C). The data are presented as M±S.E.M (n = 4). *—p<0,05, significant difference from 20% O_**2**_.

To characterise the contribution of paracrine regulation into ASC/cbHSPC interactions, we evaluated the production of 6 chemokines (G-CSF, IL-8, MCP-1, MIG, MIP-1α, MIP-1β) in the conditioned medium (CM) of ASCs monoculture and ASC/HSPCs-1 co-culture with Human Chemokine 6 Plex. Only MCP-1 and IL-8 were detected in CM from the ASC monoculture, with the concentration of both being higher at 20% O_2_ (Fig 5A). In co-culture, CM was collected when newly generated floating cbHSPCs-1 were harvested on Day 7 (96 h after washing away the unmanipulated cbMNCs). Co-culture was accompanied with considerable increase of the concentrations of both chemokines at 20% as well as at 5% O_2_ (Fig 5B), in addition, the production of MIP-1β was detected (Fig 5C).

## Discussion

Cord blood MNCs are a heterogeneous population of blood-borne cells with the vast majority of mature blood-borne elements and a small subpopulation of cbHSPC progenitors of different commitment. In this paper, we used the “physiological” approach to enrich cbHSPC fractions, as described earlier [28]. The total cbMNCs were co-cultured with ASC feeder for 3 days, after which all of the non-attached cells were removed. It was shown previously that the stroma-adhered HSPCs were the most rapidly proliferating and least committed [12,32]. Among the attached cbHSPCs, we detected both mature blood-borne cells and HSPCs of different commitments. Upon further co-culturing of attached cells, mature MNCs disappeared bit by bit, and the population of cbHSPCs was increased due to the appearance of blasts. Besides, the population of floating CD45^+^ haematopoietic cells appeared and increased above the stromal sub-layer, with their numbers being similar at 5% and 20% O_2_. In addition, we detected CAFC-like areas after 2 weeks of co-culturing. These observations are in agreement with those described previously [12,31,32]. In these papers, the three compartments of HSPC distribution in respect to the stromal layer for *ex vivo* haematopoiesis were demonstrated: adhered to the stroma surface, below the stroma and floating cells.

At present, mainly immunoselected CD34^+^ cells from the bone marrow, cord blood and mobilised peripheral blood MNCs are implicated for HSPC expansion [3,10]. This approach has some drawbacks. The CD34^+^ population is very small and loss during immunoselection extraction may be fatal [33]. Additional experimental procedures along immunoselection may result in damage of a part of the selected cells. The selection of only CD34^+^ cells leaves primitive progenitors with the CD34^-^/CD133^+^ phenotype in the unmanipulated MNC suspension. There is one more very important point. Some papers revealed the uncertainty as to whether all HSCs/HPCs express CD34 [34] and whether CD34 expression by human stem cells is a reversible process [27,35]. Moreover, Koller et al. described an enrichment of the immature populations after culturing BM MNCs, suggesting that complete CD34^+^ selection might not be strictly necessary to obtain net cell expansion [36]. Lastly, successful expansion of CD34^+^ cells requires the addition of exogenous cytokines [3,10]. In this connection, our approach for HSPC enrichment at the expense of adhesion of poorly differentiated haematopoietic cells on ASCs and their further expansion in the conventional growth medium without additional cytokine supplement seems to be very promising tool.

Despite the fact that such feature of haematopoietic niche as low O_2_ is well recognised and is considered to be one of the most important mechanisms of haematopoiesis regulation, *ex vivo* hypoxic protocols are used quite rarely [1,23,24,37,38]. Nevertheless, most of them demonstrated a positive effect of hypoxia on *ex vivo* haematopoiesis despite the considerable differences in the study design.

Thus, evaluation of haematopoietic HPSC expansion (total cell number, CFU and CD34^+^ cells) in the presence of microencapsulated osteoblasts revealed a predominant increase in the estimated parameters under 5% O_2_ [24]. In Hammoud et al. [25] expansion of CD34^+^ cells from cord blood on human bone marrow MSCs at 20, 5 and 1.5% O_2_ was studied. The authors analysed only floating cells, that are, those that comply with our HSPCs-1. “Hypoxic” cells were most efficient while repopulating the bone marrow of immune-deficient mice *in vivo*. The authors supposed that the combination of the stromal feeder and low O_2_ for *ex vivo* expansion makes it possible to fully preserve and even improve the functional activity of HSPCs. According to the authors, this is confirmed by the data from CFU-analysis, when the “hypoxic” HSPCs were most abundant with CFCs. In our work, we obtained the same results in different experimental design.

Jing et al. [26] studied the effect of 0.5% O_2_ on the interaction of CD34^+^ cells from mobilised peripheral blood and human bone marrow MSCs. Earlier, the formation of three different compartments of CD34^+^ cells during 7 days of co-culture was described in detail in this lab: (1) non-adhered fraction, (2) cells that adhered to MSCs which were shining in phase-contrast (the authors called them phase-bright cells) and (3) cells under MSCs that are dark in phase contrast (phase-dim cells). The authors showed that CD34^+^ cells in these compartments are in a different functional state. Among the adhered cells, most of the proliferating ones can be found, while the majority of resting cells (CD34^+^/CD38^-^) are found under MSCs. After division, CD34^+^ cells become unfixed from MSCs and form a floating fraction. In our work, we described the same dynamics and distribution of cbHSPCs-1 (progeny of cbMNCs) on MSCs from adipose tissue. Moreover, despite the fact that in the work by Jing et al. [32] CD34^+^ cells that migrated under MSCs were not associated with the cells that could form “cobble-stone areas”, it is quite evident that these are the same CAFCs that was described earlier by Os et al. [39] and also was demonstrated in present paper. Moreover, we managed to show that floating cbHSPCs-1 represent a heterogeneous population which (if re-cultured), may again yield the cells that occupy all three of the described compartments. Back to the results of Jing et al. [26], the authors managed to show that the area under MSCs at 20% O_2_ is the poorest in O_2_. The authors speculate that this contributes to maintaining of the non-committed state of the HSPCs found there. At 0.5% O_2_, the adhesion of CD34^+^ cells to MSCs was lower, and transmigration under MSCs was higher than at 20% O_2_, which is supposed to have occurred at the expense of the increase in MSC VEGFα production using the HIF-mediated mechanism [26].

MSCs are known to produce a whole range of mediators that take part in regulation of haematopoiesis [12,40]. Among the chemokines involved in this process, special attention is paid to C-C (MCP-1, -2, -3, MIP1-α, MIP1-β, RANTES) and C-X-C (PF-4, IL-8, IP-10) chemokine families that exhibit suppressive activity against myeloid precursors. It was shown that IL-8, PF-4, IP-10, MCP-1 suppressed the proliferation of CFU-GEMM, CFU-GM and BFU-E *in vitro*, stimulated by growth factors [41]. Here, we demonstrated that in ASC monoculture MCP-1 and IL-8 concentration in CM was higher at 20% O_2_. ASC/HSPCs interaction was accompanied with a considerable increase in MCP-1 and IL-8 production, supposedly due to an increase in chemokine-synthetic activity of ASCs. The increase of both chemokines was more pronounced at 5% O_2_. The functional activity of MCP-1 and IL-8 in ASC/cbHSPCs co-culture is related to inhibition of primitive HSPC cycling, which supports the pool of non-differentiated progenitors [42,43]., Another member of the C-C family—MIP-1β, was revealed in CM of co-cultured ASC/HSPCs. This chemokine does not directly stimulate or inhibit HSPCs; however, being in excess compared with the cognate chemokine MIP-1α, this blocks MIP-1α-mediated inhibition of the proliferation of myeloid progenitors [44]. So, interactions between ASCs and cbHSPCs entails simultaneous increase in cytokines providing negative and positive regulation of self-renewal and differentiation of cbHSPCs, which generally ensures a balanced paracrine profile that supports both primitive and committed haematopoietic progenitors.

Thus, the combination of unfractionated cbMNCs and human ASCs enables the selection of functionally active cbHSPCs, the further expansion of which without exogenous cytokines provides enrichment with CD34^+^ cells. ASCs efficiently support the viability of cord blood haematopoietic progenitors of different commitment at standard and tissue-related O_2_ levels at the expense of direct and paracrine cell-to-cell interactions. A reduced O_2_ concentration makes it possible to increase the share of CD34^+^ cells in the cbHSPC population and ensure the predominant development of certain haematopoietic lineages. These data are of importance both from the viewpoint of fundamental physiological haematopoiesis mechanisms and in connection with the importance of directed *ex vivo* cbHSPC expansion for the needs of regenerative medicine.
